# A Prospective Study of the Role of Tranexamic Acid in Postoperative Bleeding and Seroma in Modified Radical Mastectomy

**DOI:** 10.7759/cureus.92339

**Published:** 2025-09-15

**Authors:** Mangalapalle MadhuBabu, Shyam Lal, Swagata Bramhachari

**Affiliations:** 1 Department of General Surgery, All India Institute of Medical Sciences, Bhopal, Bhopal, IND

**Keywords:** breast cancer, modified radical mastectomy, postoperative bleeding, seroma, tranexamic acid

## Abstract

Background: Modified radical mastectomy (MRM) is a common surgical procedure for breast cancer, but is frequently complicated by seroma formation and postoperative bleeding, leading to morbidity and delayed recovery. Tranexamic acid (TXA), an antifibrinolytic agent, has shown efficacy in reducing blood loss across surgical specialties. This study evaluates its role in minimizing postoperative bleeding and seroma formation following MRM.

Methods: A non-randomized interventional study was conducted at the Department of General Surgery, All India Institute of Medical Sciences (AIIMS), Bhopal, from April 2023 to August 2024. Fifty female breast cancer patients undergoing MRM were enrolled and allocated to two groups: TXA group (n=25) and non-TXA group (NTXA, n=25). The TXA group received 1 g IV TXA at anesthesia induction, followed by 500 mg orally every eight hours for five days. Demographic variables, comorbidities, intraoperative blood loss, and postoperative parameters (daily and total drain output, drain duration, seroma formation, wound healing, and hospital stay) were recorded. Data were analyzed using IBM SPSS Statistics, version 25 (IBM Corp., Armonk, USA), with p < 0.05 considered significant.

Results: Both groups were comparable with reference to age, BMI, and clinical stage. Mean intraoperative blood loss was significantly lower in the TXA group (84.8 ± 32.06 ml) compared to the NTXA group (106.4 ± 37.95 ml; p = 0.04). Although total drain output (p = 0.33) and drain duration (p = 0.36) were not significantly different, daily drain output was significantly lower in the TXA group from Day 4 onwards (Day 4, p = 0.03; Day 5, p = 0.04; Day 6, p = 0.02; Day 7, p = 0.02). Seroma formation after drain removal occurred in 3 (12%) of TXA patients versus 5 (20%) in the NTXA group, but this was not statistically significant (p = 0.70). The mean hospital stay was significantly shorter in the TXA group (6.13 ± 2.29 days) than in the NTXA group (7.40 ± 1.84 days; p < 0.01).

Conclusion: Tranexamic acid significantly reduces intraoperative blood loss and late postoperative drain output in MRM patients. However, it does not significantly decrease overall seroma incidence or drain duration. Importantly, TXA use was associated with a shorter hospital stay. Larger randomized controlled trials are warranted to validate its role in reducing postoperative complications following MRM.

## Introduction

Breast cancer continues to be a significant health challenge worldwide, and surgery forms the cornerstone of treatment. Modified radical mastectomy (MRM) is still among the most frequently performed operations, particularly in countries such as India, where late-stage presentation is common [1]. One of the most troublesome complications following MRM is postoperative seroma, with reported incidence varying widely from 3% to 90%. Seroma is not just a minor inconvenience; it is often associated with pain, repeated aspirations, prolonged drainage, delayed wound healing, increased clinic visits, and slower recovery [2].

Over the years, several measures have been attempted to reduce seroma formation, including closed-suction drainage, compression garments, and flap fixation. Yet, outcomes have been inconsistent, and seroma remains a frequent and unresolved morbidity [3]. While obliteration of dead space may help, it is not routinely adopted because it adds to the operative time and demands additional expertise [4].

Tranexamic acid (TXA), an antifibrinolytic drug, prevents the conversion of plasminogen to plasmin, thereby stabilising fibrin clots. Its effectiveness in reducing perioperative bleeding is well documented across many surgical specialties [5]. In breast surgery, TXA has been reported to decrease blood loss and transfusion requirements, and it is hypothesised to reduce seroma by limiting oozing and early exudation from mastectomy flaps [6]. Although multiple studies have investigated the use of tranexamic acid in breast surgery, there remains no consensus on its optimal use. The route of administration, appropriate dosage, and timing of administration vary widely across studies, and standardized protocols have yet to be established [7].

Given the high burden of seroma after MRM and the biologic plausibility that TXA could reduce late fluid accumulation, procedure-specific evidence is warranted. Therefore, this prospective study was designed to assess the role of TXA in reducing postoperative drainage and seroma formation after MRM and generate practical data relevant to routine practice in resource-limited settings.

## Materials and methods

Study design and setting

This was a non-randomized interventional study conducted from April 1, 2023, to August 31, 2024 (18 months) at the Department of General Surgery, All India Institute of Medical Sciences (AIIMS), Bhopal, after approval from the Institutional Human Ethics Committee (LOP-AIIMS/BPL/IHECSR/JULY/22/PG/05).

Participants

Eligible participants were female patients aged ≥18 years with operable breast carcinoma undergoing modified radical mastectomy, including those receiving neoadjuvant therapy. Exclusion criteria were metastatic disease, history or high risk of thromboembolism (including previous history of deep vein thrombosis/pulmonary embolism, known thrombophilia like Factor V Leiden, protein C/S deficiency, antithrombin III deficiency, antiphospholipid syndrome, heart failure, nephrotic syndrome, inflammatory bowel disease), current anticoagulant therapy, refusal of consent, pregnancy-associated breast carcinoma, being unfit for surgery, male sex, or patients planned for breast-conserving surgery. Written informed consent was obtained from all participants.

Sample size and group allocation

Patients were assigned to two non-randomized cohorts based on the operating surgeon: those operated by the investigators (guide/co-guide) formed the TXA group; those operated by other surgeons formed the non-TXA (NTXA) group. Sample size was calculated using Kelsey’s method for two proportions, referencing prior seroma incidences (TXA 19.1% vs. NTXA 32.6%) [8], with 80% power, 95% confidence, and 15% anticipated dropout, yielding 50 patients (25 per group).

Intervention

**Figure 1:**
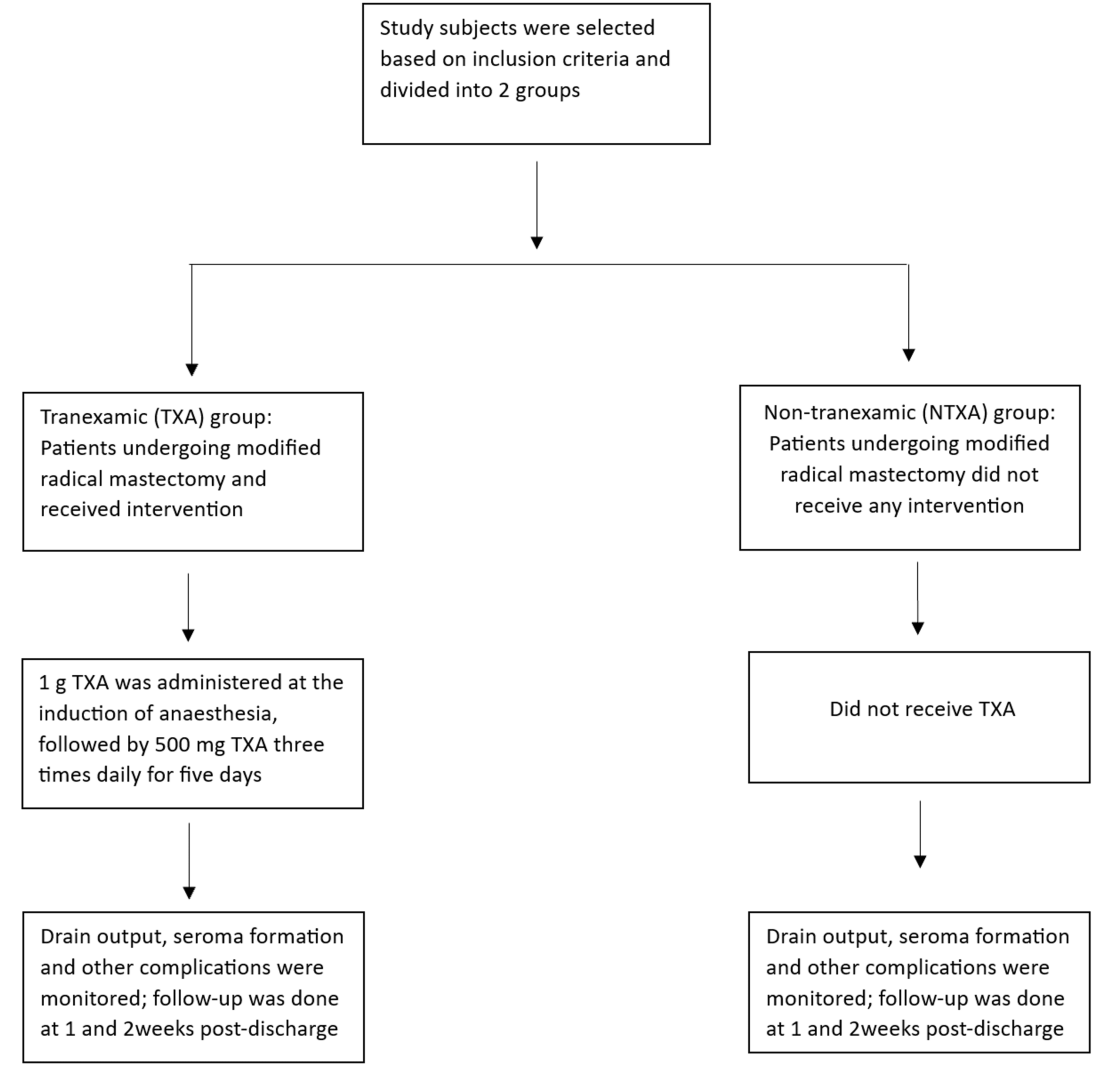
CONSORT diagram of the study TXA: tranexamic acid; CONSORT: Consolidated Standards of Reporting Trials

The TXA protocol comprised a single intravenous dose of 1 g tranexamic acid diluted in 100 ml normal saline at induction of anesthesia, followed by 500 mg oral TXA every eight hours for five days post-operatively [9]. Controls received no TXA. The CONSORT diagram is shown in Figure 1.

Operative technique and perioperative care

All patients underwent standard MRM via a Stewart elliptical incision with elevation of skin flaps to defined anatomical boundaries, axillary dissection with nerve preservation, and placement of a two-limb suction drain of size 16 Fr, one limb in the axilla and the other beneath the skin flaps over the chest wall, followed by layered closure. Specimens were sent for histopathology.

Outcomes and measurements

Primary variables included intra-operative bleeding measured using the amount collected in the suction canister (after subtracting irrigation fluid) and the weight of soaked surgical sponges/gauze (with 1 g weight gain taken as equivalent to 1 mL of blood), postoperative bleeding measured by daily drain output and total output and seroma formation after drain removal. Secondary outcomes were duration of drain, postoperative hospital stay, flap necrosis, surgical site infection (SSI), and other wound-related complications. Drain output was quantified using calibrated collection, and complications were prospectively recorded. Participants were reviewed at one and two weeks after discharge.

Follow-up

All patients were followed up in the outpatient clinic one and two weeks post-discharge to assess seroma formation and wound outcomes.

Statistical analysis

Data were entered in Microsoft Excel (Microsoft Corporation, Redmond, USA) and analyzed using IBM SPSS Statistics, version 25 (IBM Corp., Armonk, USA). Normality was assessed with the Kolmogorov-Smirnov test. Between-group comparisons were done using the unpaired t-test or Mann-Whitney U test for quantitative variables and chi-square or Fisher’s exact test for categorical variables. A two-sided p < 0.05 was considered statistically significant.

## Results

Demography and baseline characteristic features

The patients in both groups were comparable in terms of mean age, BMI and comorbidities with no statistically significant difference (p > 0.05), ensuring that these factors did not influence the study outcomes. Based on TNM (tumour, node, metastasis) classification, the disease's clinical stage was evenly distributed, with no significant variation in the proportion of early-stage (Stages I and II) and advanced-stage (Stage III) cases between the groups. The hormonal status, including estrogen receptor (ER), progesterone receptor (PR), and HER2 expression, was similar across both groups, ensuring that differences in hormone receptor profiles did not introduce bias (Tables 1-3).

**Table 1:** Baseline characteristics of patients TXA: tranexamic acid; NTXA: non-TXA

Variable	TXA, mean ± SD	NTXA, mean ± SD	p-value
Mean age (years)	51.40 ± 11.20	51.88 ± 10.87	0.87
BMI (kg/m²)	22.90 ± 2.52	22.87 ± 2.42	0.96
Tumour size (cm)	4.30 ± 1.52	4.00 ± 1.38	0.52

**Table 2:** Clinical staging of patients TXA: tranexamic acid; NTXA: non-TXA

Clinical stage	TXA, n (%)	NTXA, n (%)	p-value
I	4 (16%)	5 (16%)	0.98
IIA	7 (28%)	8 (32%)	0.98
IIB	9 (36%)	7 (28%)	0.76
IIIA	4 (16%)	4 (12%)	1.00
IIIB	1 (4%)	1 (4%)	1.00

**Table 3:** Hormonal receptor status TXA: tranexamic acid; NTXA: non-TXA

Variable	TXA, n (%)	NTXA, n (%)	p-value
ER positive	4 (16.0%)	10 (40.0%)	0.11
PR positive	7 (28.0%)	6 (24.0%)	1.0
Her2neu positive	8 (32.0%)	12 (48.0%)	0.38

Comparison of operation time and blood loss (ml)

Intraoperative blood loss was significantly lower in the test group (84.8 ± 32.06 ml) compared to the control group (106.4 ± 37.95 ml), with a statistically significant p-value of 0.04, indicating that tranexamic acid effectively reduces blood loss during surgery (Table 4).

**Table 4:** Operation time and blood loss (ml) between both groups TXA: tranexamic acid; NTXA: non-TXA

	TXA, mean ± SD	NTXA, mean ± SD	p-value
Blood loss (ml)	84.8 ± 32.06	106.4 ± 37.95	0.04
Operation time duration (min)	151.80 ± 31.18	144.4 ± 32.41	0.27

Postoperative drain output

**Figure 2:**
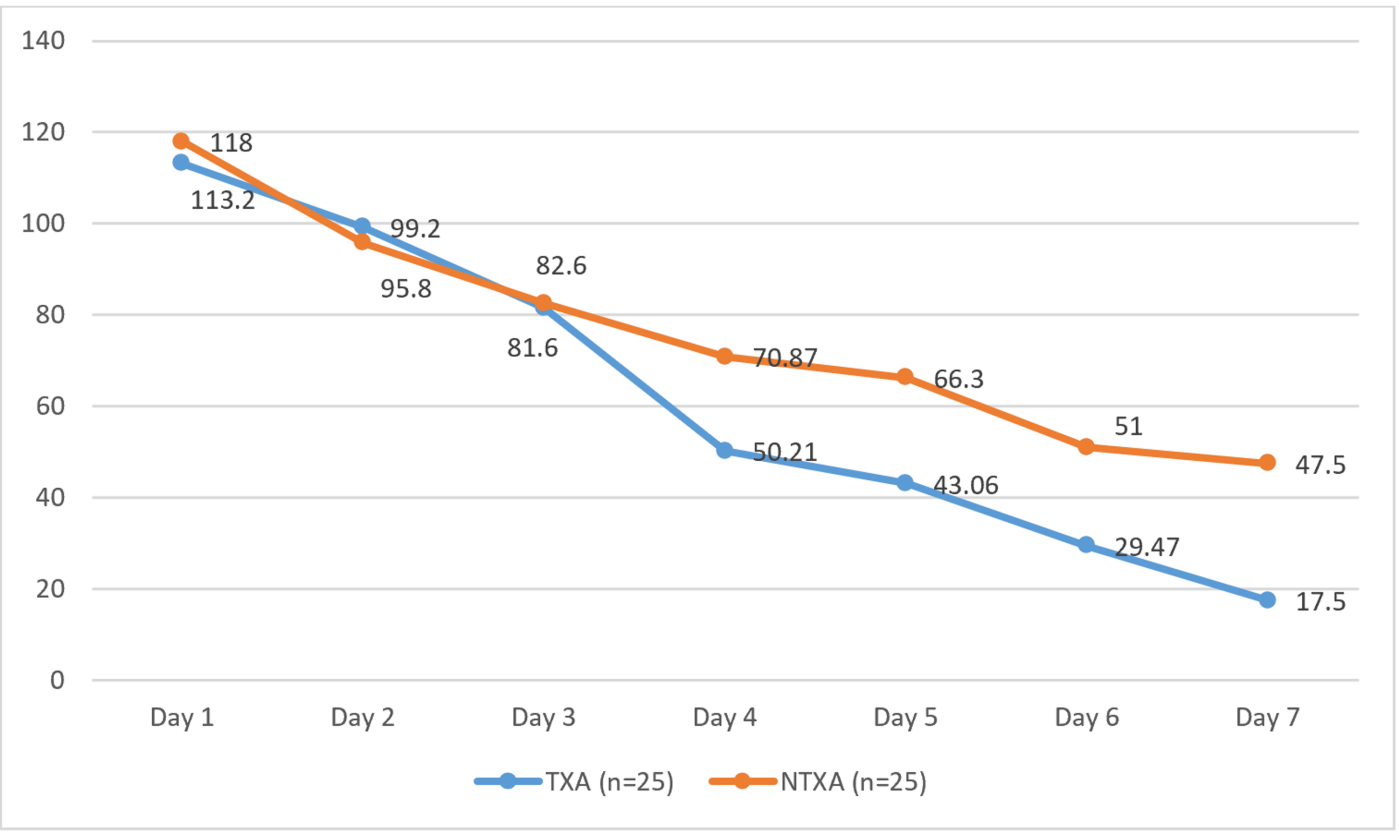
A comparison of postoperative drain output for both groups at different intervals TXA: tranexamic acid; NTXA: non-TXA

The comparison of daily drain output between the TXA and NTXA groups showed no significant difference in the first three days (p > 0.05). However, from Day 4 onward, the TXA group exhibited a significant reduction in drain output compared to the NTXA group (p = 0.03, 0.04, 0.02, and 0.02 on Days 4-7, respectively), suggesting its role in minimizing seroma formation. Although total drain output was lower in the TXA group (500.71 ± 227.25 ml) than in the NTXA group (662.14 ± 377.18 ml), this difference was not statistically significant (p = 0.33). These findings indicate that tranexamic acid may help reduce postoperative drainage (Table 5, Figure 2).

**Table 5:** Postoperative drain output at different intervals TXA: tranexamic acid; NTXA: non-TXA

Drain output	TXA, mean ± SD	NTXA, mean ± SD	p-value
Day 1	113.2 ± 54.67	118.0 ± 81.03	0.72
Day 2	99.20 ± 50.47	95.80 ± 65.34	0.59
Day 3	81.60 ± 50.94	82.60 ± 71.80	0.61
Day 4	50.21 ± 41.81	70.87 ± 37.52	0.03
Day 5	43.06 ± 44.33	66.30 ± 50.99	0.04
Day 6	29.47 ± 24.77	51.0 ± 39.56	0.02
Day 7	17.50 ± 7.90	47.50 ± 32.84	0.02
Total drain output (ml)	500.71 ± 227.25	662.14 ± 377.18	0.33

Postoperative complications

The comparison of postoperative outcomes between the TXA and NTXA groups indicates that tranexamic acid had no significant impact on duration of drain, SSI, flap necrosis and seroma formation, as there was no statistically significant difference between the groups. However, postoperative hospital stay was significantly shorter in the TXA group (6.13 ± 2.29 days) compared to the NTXA group (7.40 ± 1.84 days), with a significant p-value of <0.01, indicating a potential benefit in faster recovery and earlier discharge.

## Discussion

Seroma formation post-MRM is defined as an exudative fluid collection that develops postoperatively underneath the mastectomy flaps and in the axillary dead space. This leads to increased patient discomfort, risk of infection, flap complications, and delayed wound healing [10]. The etiopathogenesis of seroma formation is not well understood. Although various interventions have been proposed to minimize seroma formation, including the use of compression bandages, suction drains by Divino et al. (2000), flap fixation with sutures by O’Dwyer et al. (1991), sclerotherapy by Nichter et al. (1983), and fibrin glue by Jain et al. (2004), Moore et al. (2001), Johnson et al., 2005, and Dinsmore et al. (2000), to eliminate dead space, none of them proven to be effective in controlling seroma formation [11-17].

A comparison of total drain output and drain duration across studies showed the effects of tranexamic acid on postoperative drainage. Gonga et al. (2015) [18] reported a significant reduction in both total drain output and duration of drain removal in the TXA group. Eldesouky et al. (2019) [19] demonstrated a significant difference in the duration of drain removal, though the reduction in total drain output was not statistically significant. Weissler et al. (2022) [20] also observed a reduction in drain duration in patients receiving TXA, but the difference did not reach statistical significance. In our study, although the drain output was lower in the TXA group compared to the non-TXA group, the difference was not statistically significant, nor was the duration of drain removal (Table 6).

**Table 6:** Comparision of drain output and duration with other studies TXA: tranexamic acid; NTXA: non-TXA

Authors	Year	Study design	n (TXA/NTXA)	Total drain output (mean ± SD), TXA group	Total drain output (mean ± SD), NTXA group	p-value (drain output)	Duration of drain (mean ± SD), TXA group	Duration of drain (mean ± SD), NTXA group	p-value (drain duration)
Gonga and Goyal [18]	2015	Prospective	25/25	781.4 ± 248.64	1023 ± 196.3	0.001	10.2 ± 2.12	13.72 ± 2.40	0.001
Eldesouky et al. [19]	2019	Prospective	65/50	798 ± 107.3	1067.1 ± 188.6	0.308	9.85 ± 1.66	11.67 ± 1.9	<0.005
Weissler et al. [20]	2022	Retrospective	257/128	-	-	-	6.5 ± 2.3	7.3 ± 4.5	0.1
Present study	2023	Prospective	25/25	500.71 ± 227.25	662.14 ± 377.18	0.33	8.0 ± 3.34	7.76 ± 3.87	0.36

The data from various studies, including the present study, suggest that the use of tranexamic acid does not have a significant impact on seroma formation after drain removal or wound-related complications. In studies by Eldesouky et al. [19], Weissler et al. [20], Lohani et al. [8] and Ausen et al. [21], the incidence of seroma and wound-related complications was comparable between the TXA and NTXA groups, with p-values indicating no statistically significant difference. Similarly, in the present study, seroma formation after drain removal was observed in 12% of patients in the TXA group and 20% in the NTXA group (p = 0.70), while wound-related complications occurred in 12% and 20% of patients in the TXA and NTXA groups, respectively (p = 0.70), reinforcing the finding that TXA does not significantly reduce these postoperative complications (Table 7).

**Table 7:** A comparison of seroma and wound-related complications TXA: tranexamic acid; NTXA: non-TXA

Authors	Year	Study design	n (TXA/NTXA)	Seroma after drain removal, TXA group, n (%)	Seroma after drain removal, NTXA group, n (%)	p-value	Wound-related complications, TXA group, n (%)	Wound-related complications, NTXA group, n (%)	p- value
Eldesouky et al. [19]	2019	Prospective	65/50	8 (12.3)	6 (12)	0.508	3 (4.6)	1 (2)	0.790
Weissler et al. [20]	2022	Retrospective	257/128	10 (2)	5 (2)	0.94	17 (2.2)	14 (2.7)	0.94
Lohani et al. [8]	2020	Prospective	47/46	9 (19.1)	15 (32.6)	0.13	2 (4.3)	4 (8.7)	0.13
Ausen et al. [21]	2020	Prospective	52/56	N/A	N/A	N/A	5 (9.6)	6 (10.7)	1
Present study (2023)	2023	Prospective	25/25	3 (12)	5 (20)	0.70	3 (12)	5 (20)	0.70

Limitations

This study has several limitations, including a small sample size (n = 50), which may limit the statistical power to detect significant differences. The non-randomized, single-center design introduces the possibility of selection bias and may limit the generalizability of the findings. Furthermore, the short follow-up period may not capture long-term complications. A larger, multi-center, randomized, double-blind study would be needed to provide more definitive evidence on the role of tranexamic acid in reducing postoperative complications after modified radical mastectomy.

## Conclusions

This study demonstrates that tranexamic acid effectively reduces both intraoperative blood loss and postoperative drain output, and decreases the length of hospital stay. However, it did not show a statistically significant reduction in overall seroma formation or wound complications. Further studies with larger, more robust designs are needed to confirm these findings and establish the clinical benefits of tranexamic acid in breast cancer surgery.
